# State-of-the-art optimization methods for short-term mine planning

**DOI:** 10.12688/f1000research.152986.1

**Published:** 2024-09-30

**Authors:** Moise Kambala Malundamene, Nasib Al Habib, Saâd Soulaimani, Khalil Abdessamad, Hooman Askari-Nasab

**Affiliations:** 1 Mining Engineering, Ecole Nationale Superieure des Mines de Rabat, Agdal, Rabat-Sale-Zemmour-Zaer, Morocco; 2 Civil and Environmental Engineering, University of Alberta, Edmonton, Alberta, t6E 2J7, Canada; 3 Mohammed VI Polytechnic University (UM6P), Geology & Sustainable Mining Institute (GSMI), Lot 660, Hay Moulay Rachid, Ben Guerir, Morocco

**Keywords:** Open pit mines, Mining planning optimization, Short-term mine planning, operational planning

## Abstract

Maintaining short-term planning aligned with the ultimate long-term plan is challenging. This requires many details to be modelled on a daily or weekly basis to reach this target. Short-term planning is more challenging than medium- and long-term planning because it deals with uncertainties, which increases the gap between medium- and long-term plans. Short-term mine planning teams are expected to identify and manage potential risks to mitigate them, and eventually achieve the long-term objective of maximizing the Net Present Value (NPV). Very few studies have identified the problems that exist in short-term mine planning and provided technical solutions to overcome them for open-pit mines. One of the complexities associated with short-term planning is the creation of polygons or mining cuts by clustering before optimizing and scheduling the plan to reduce the computational expense of mine planning models.

The primary objective of this study is to review the latest papers describing short-term mine planning challenges and technical solutions proposed to optimize mine planning for open-pit mines.

## 1. Introduction

One of the aims of short-term production scheduling is to generate a plan that meets the production and grade targets according to the budget of the long-term mine plan ( Chanda and Wilke 1992). Extensive modelling of daily or weekly activities is required to achieve the production target and grade requirements. These targets comply with equipment utilization, processing capacities, recovery, and processing requirements managed in short-term planning ( Blom et al. 2019). Short-term mine planning is more challenging because it mostly deals with many uncertainties on a daily basis, such as geological uncertainties ( Tabesh and Askari-Nasab 2019), operational and dispatching uncertainties ( Upadhyay et al. 2015; Upadhyay and Nasab 2017; Upadhyay and Askari-Nasab 2018; Mohtasham et al. 2021; Nelis and Morales 2022) and economic uncertainties ( Osanloo and Rahmanpour 2017).

The gap between medium- and long-term planning in terms of achieving the production target may reach more than 50%, which can be a costly reconciliation in the long term ( Kahraman and Dessureault 2011). Thus, short-term mine planners with management teams are requested to identify and manage risks to mitigate them and adhere able to stick to the Life-of-Mine (LOM) plan.

While there are quite a few literature reviews on long-term planning ( Osanloo et al. 2008; Newman et al. 2010; Kozan and Liu 2011; Lamghari 2017; Blom et al. 2019; Fathollahzadeh et al. 2021), very few have been carried out for short-term planning to the best of our knowledge ( Blom et al. 2019; Habib et al. 2023). These articles focused mostly on short-term planning optimization without developing the optimization aspect of mining polygons, a basic element for short-term mine planning ( Nelis and Morales 2022). Later, Fathollahzadeh et al. (2021) and Habib et al. (2023) presented main methodologies, such as deterministic, heuristic, metaheuristic, and stochastic methods. used for short-term planning optimization. Table 1 summarizes the main review papers on mining planning ( Fathollahzadeh et al. 2021).

**Table 1.** Review articles for the mining planning (long-term and short-term).

Article review	Mining methods	Scope
( Osanloo et al. 2008)	Open pit mine	Long-term
( Newman et al. 2010)	Underground *&* open pit mine	Long-term *&* short-term
( Mousavi et al. 2016a) ( Kozan and Liu 2011)	Underground *&* open pit mine	Long-term *&* short-term
( Lamghari 2017)	Open pit mine *&* oil field	Long-term *&* short-term
( Blom et al. 2019)	Open pit mine	Short-term
( Fathollahzadeh et al. 2021)	Open pit mine	Long-term
( Habib et al. 2023)	Open pit mine	Short-term
This article review	Open pit mine	Short-term

### 1.1 Relation between Long-term and short-term Mine planning

Good mining planning is the basis for the best financial results for a mining company. Short-term mine planning is set up to align with the company strategy. One of the main differences between short-term mine planning and long- and medium-term mine planning is time discretization. Short-term planning should be performed with a granularity of less than one year. This granularity can be extended for up to two years ( Blom et al. 2019). Short-term mine planning is required to achieve the goals set by a long-term plan by designing push-backs. Generally, the objective is to generate the highest cash flow value and Net Present Value (NPV) over the LOM plan ( Juarez et al. 2014; King 2014; Otto and Musingwini 2020) while minimizing costs and respecting production targets within the LOM plan ( Jewbali and Dimitrakopoulos 2018; Noriega and Pourrahimian 2022). This involves incorporating more sophisticated mathematical analysis and advanced geostatistics techniques, performed by a specific and adequate softwares (for example: Datamine RM, Datamine UG, Snowden and NPV), However, different models and algorithms, are applied to enhance performance and optimize short-term operations, ultimately contributing to improved production targets, cost reduction, and increased efficiency. Short-term mine planning deals with the equipment and resources allocated on a monthly, weekly, daily, or shift-by-shift basis under the control of the LOM ( Noriega and Pourrahimian 2022). Thus, the success of truck fleet and shovel allocation to mining face areas depends strongly on the best dispatching strategy for fleet management. Models and algorithms for the fleet management systems have been reviewed by Moradi Afrapoli and Askari-Nasab (2019).

Few papers have described short-term planning with granularity in days, weeks, and months. Table 2 specifies the main difference between short- and long-term mining planning, taking into account the discretization time, block model characteristics, design, mining precedence, cost, and equipment. These plans take into account the objective functions and constraints to be considered and the level of details for mine operations to be considered and modelled. This includes constraints specific to each mine, their capacities, block sequencing, blending, plant requirements, and pit slopes ( Caccetta and Hill 2003; Askari-Nasab et al. 2010; Marques et al. 2013; Badiozamani and Askari-Nasab 2014; Blom et al. 2019).

**Table 2.** Main difference aspects between long-term and short-term mine planning.

Aspects	Long-term mine plan	Short-term Mine plan
Discretization of time	Quarterly to yearly often for medium term or more for long-term,	Shift-to-shift, daily, Weekly, Monthly
Block model	Millions of equally sized blocks according the grade (High Grade, Low Grade, Waste) for different destination (Plant, stockpile and waste dump) respectively	Set or portions of blocks with irregularly shaped according the grade (High Grade, Low Grade, Waste) for different destination (Plant, stockpile and waste dump) respectively
Geotechnical	Global pit stability with a safe and optimal overall pit angle, and pit monitoring	Bench face angle stable and suitable for mining operations
Design	Pit Global design and pushback respecting pit slope (overall angle)	Pushbacks designs, faces availables,
Mining precedence’s	Blocks directly above to be extracted before a block below according the pit slope wall	Accessing blocks from the mining faces
Cost	Maximization of Net Present Value (NPV)	maximizing equipment utilization and Minimization costs or losses (Rehandling …)
Equipment’s	Fleet size available (Number of Trucks)	Modelling of individual of equipment available (Drill rigs, shovels and trucks …

**Figure 1.**
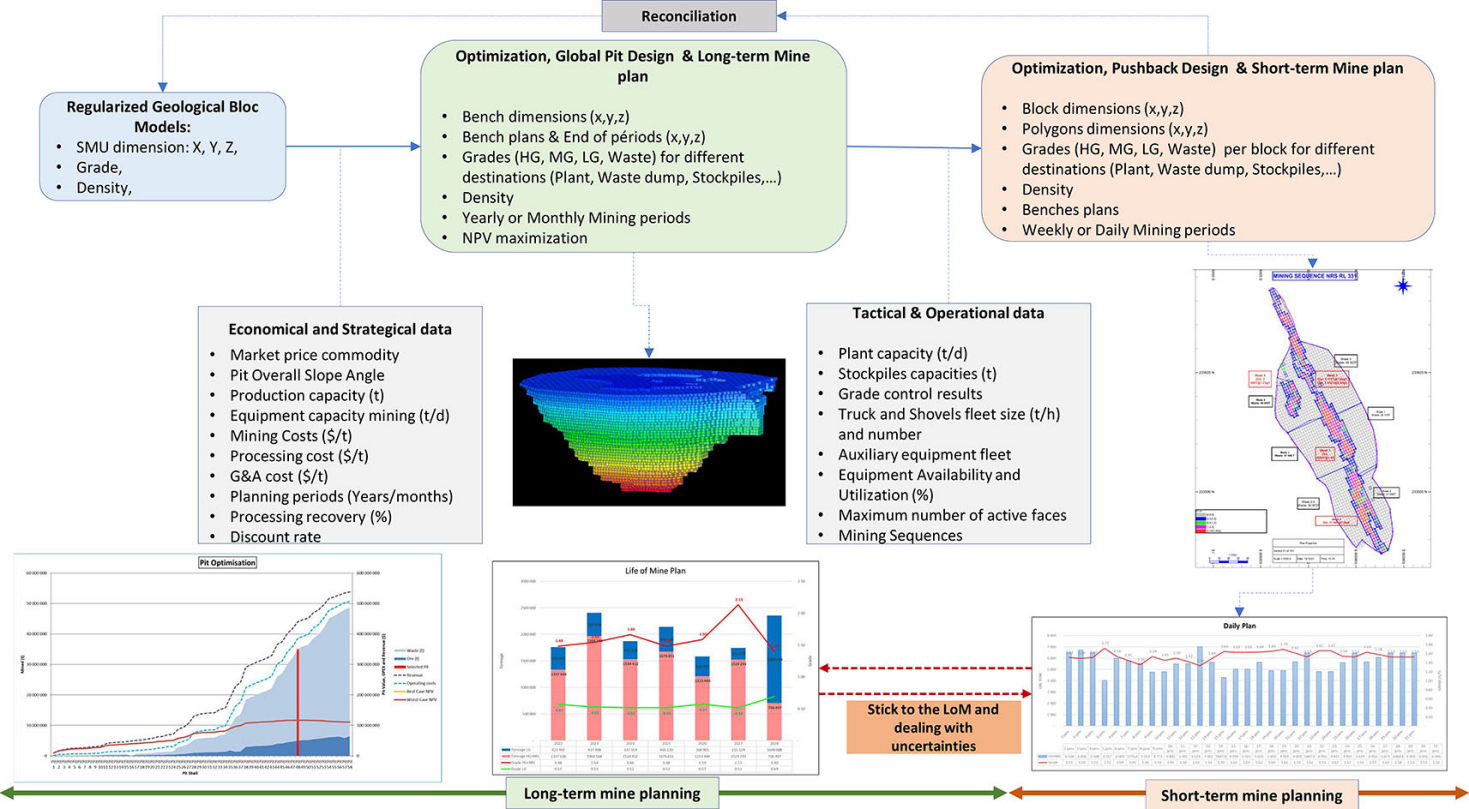
Relation between Long-term and Short-term Mine planning and area of the study.

Figure 1 illustrates the relationship between long-term and short-term mine plans based on the scope area limit of this review article.

### 1.2 Research gaps for short-term planning

In the last decade, some studies have discussed short-term mine planning problems and technical solutions on a monthly basis ( Askari-Nasab et al. 2010; Tabesh and Askari-Nasab 2013; Blom et al. 2019; Salman et al. 2021; Menezes and dos Santos Corrêa 2022).

However, short-term mine planning optimization for open-pit mines remains underexplored.

Short-term planning models must be solved quickly because of the dynamic nature of mining operations. Mining polygon optimization reduces the computational expenses of mine planning models by reducing the number of variables involved. Therefore, more research is required to develop more efficient mathematical models. The primary goal of this study is to review existing publications on short-term mine planning and mining polygon optimization methods to galvanize researchers to work on the quicker solvability of short-term mine planning models.

The first step focuses on the techniques used to create and optimize mining polygons, which is an important step in scheduling. In the second step, we will focus deeply to review different approaches for short-term mine planning optimization. Before concluding and stating the next orientation of this work, we will discuss all approaches reviewed on the recent improvements for overcoming uncertainties in short-term mining planning.

## 2. Scope of the research

The aim of this research is to understand the current state-of-the-art optimization methods for short-term mine planning. The objective of this review article is to focus on the following aspects of short-term planning:

• Dig-limit optimization and Mining polygons,
• Short-term production scheduling optimization,

The first point will help to identify the main methods for creating mining polygons for short-term mining planning. The second point is related to short-term scheduling with suitable mining cuts for short-term planning optimization.

This research will use the available scientific database (Scopus journals, Sciences Direct, Taylor and Francis, Google Scholar) to review the retrieved papers until June 2023.

Underground operations, Geotechnical and Processing aspects are also beyond the scope of long-term planning.

## 3. Literature review of methods to perform mining polygons

Mining polygon creation is the most critical task to be performed after optimization and design. This heavy task is the result of a grade control program, and is often performed manually by mining planners ( Nelis and Morales 2022). More research has been carried out since the last decade for automatically generating mining cuts with algorithms; however, unfortunately, none of them have been applied at the industrial scale, and all these studies have been conducted at the academic level.

In the literature, we can identify two main ways to perform mining polygons for short-term mine planning, as follows:

• Dig-limit optimization is used to define the ore-waste limit ( Norrena and Deutsch 2001; Richmond and Beasley 2004; Ruiseco et al. 2016; Sari and Kumral 2018; Nelis and Morales 2022);
• Clustering techniques with agglomeration of similar bloc properties ( Askari-Nasab et al. 2010; Tabesh and Askari-Nasab 2013; Nelis and Morales 2022);

### 3.1 Dig-limits optimizations

The dig-limit optimization approach consists of defining the best possible limits between ore waste on a bench basis to ensure the profitability of mining operations based on the definition ( Sari and Kumral 2018). The definition of polygons depends on the level of bloc model preparation (grade control program), which affects the grade uncertainty ( P et al. 2002; Neufeld et al. 2003; Richmond and Beasley 2004; Vasylchuk and Deutsch 2019).

Generally, in mining industry practice, the dig-limit is delimited manually by a geologist after obtaining the grade control results before blasting ore. After delimitation of the ore according to the cut-off grade, the geologist and mining teams can decide the destination of the material to be blasted: ore to the plant for high grade (HG), ore to the stockpile for the low-grade (LG), and waste to the waste dump.

Several techniques have been used to perform dig-limits and ore polygons ( Table 3). Sari and Kumral (2018) used Mixed Integer Linear Programming Richmond (2007), used local search algorithms Richmond and Beasley (2004), used a heuristic approach with a floating cone algorithm to find an optimal solution for the grade control problem. To determine the optimal final destination for materials (ore or waste), ( Vasylchuk and Deutsch 2019) also used a simple heuristic approach for mineable dig-limit optimization considering the excavation constraint while maximizing profit. Heuristic algorithms are known to solve problems faster and more efficiently, while sacrificing optimality and accuracy.

**Table 3.** Literature review of mining polygons modelling.

	Dig-limit polygons Approaches	Authors	Results
Clustering Techniques	Hierarchical clustering	( Tabesh and Askari-Nasab 2011; Tabesh and Askari-Nasab 2013)	Cluster shapes not controlled and non-practical clustering patterns and similarity index takes only one element into account
		( Tabesh and Askari-Nasab 2019)	Cluster shapes-controlled similarity index takes into account multi elements and grade uncertainty
	K-Means Clustering	( Salman et al. 2021)	Mineable cluster shapes, high destination homogeneity, rock unity. Possibility to extend complex multi-element, multi-destination deposits and to incorporate grade uncertainty
Dig-limit optimizations	Genetic algorithms	( Ruiseco et al. 2016)	The dig-limits constraints equipment incorporated, performs better than simulated annealing and works with multiple rock types, processes and metals
	Simulated annealing	( Norrena and Deutsch 2001; P et al. 2002; Neufeld et al. 2003)	Dig-limits constraints incorporated, maximizes the profit and penalizes smaller angles of operation taking into account the digability. Solution space search algorithm moves toward non-improving solutions with a certain probability.
	Grade control strategy of SMUs using Mixed Integer Linear Programming	( Sari and Kumral 2018; Nelis et al. 2022; Nelis and Morales 2022)	Dig-limits constraints incorporated by Sari and Kumral (2018), cut-off grades not used and the model minimizes the loss associated with misclassification of SMU’s
	Greedy search: Feasibility grade control Floating Circle algorithm	( Wilde and Deutsch 2007)	Requires an initial solution and attempts to optimize the profit iteratively by re-arranging the form of blocks accumulated into units.
	Local search algorithm	( Richmond and Beasley 2004)	Dig-limits constraints incorporated, minimize ore loss and mining dilution by using a payoff function per block
	Heuristic approach: Floating cone algorithm	( Richmond and Beasley 2004; Vasylchuk and Deutsch 2019)	Dig-limits constraints incorporated, minimize ore loss and dilution. Multiple ore types and objective functions taken into account to maximize the net present value (NPV), no guarantee of optimality. Dig-limit optimization in 2-D

Ruiseco et al. (2016) used genetic algorithms to automatically generate a dig-limit considering the selectivity of the fleet for the Selective Mining Unit (SMU) while maximizing profitability. In this approach, the dig-limit is considered as a chromosome and each SMU is considered as a gene. The first population is generated by a random set of feasible dig-limits that are combined with others to reach the optimized dig-limit. The genetic algorithm for dig-limits is better than manual methods; this algorithm is a near-optimal solution Sari and Kumral (2018).

Simulated annealing was applied by Isaaks et al. (2014) using the minimum expected loss method to find the optimal dig-limits constrained by a minimum width. Neufeld et al. (2003) used a semi-automatic algorithm for the dig-limit with the initial polygons by the user. Norrena and Deutsch (2001) and P et al. (2002) proposed an algorithm that optimizes dig-limit boundaries with maximum profit, taking into consideration the equipment’s limitations (digability).

The Heuristic approach was used by Vasylchuk and Deutsch (2019), who proposed a 2-D optimization dig-limit with multiple locations and destinations by maximizing profits. Contrary to Norrena and Deutsch (2001) and P et al. (2002), this algorithm uses a rectangular form of and for the dig limit, and there are no smoothing operations after their generation.

### 3.2 Clustering algorithms

Clustering algorithms attempt to identify groups or clusters of elements with the same properties in datasets. In short-term planning, clustering is used to aggregate blocks with the same properties to generate mining cuts. A mining cut is a SMU that generates a practical mining schedule ( Eivazy and Askari-Nasab 2012).

**Figure 2.**
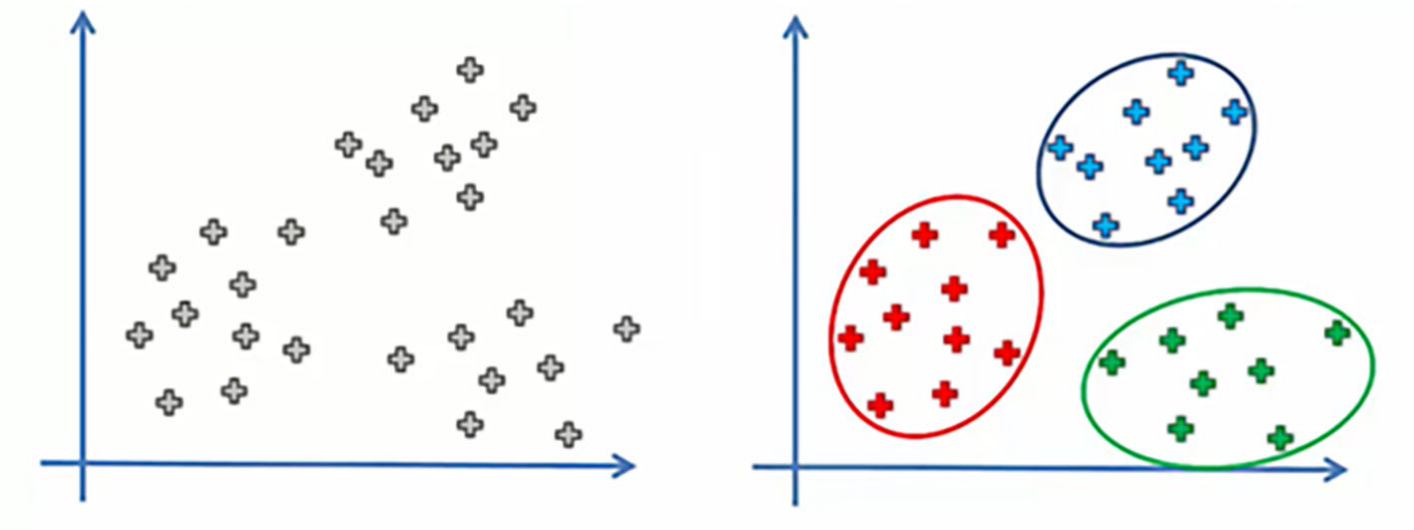
Before and after clustering data.

Tabesh and Askari-Nasab (2013) described clustering as the process of combining related things in a manner that maximizes intra-cluster similarity and inter-cluster dissimilarity ( Figure 2).

Clustering algorithms for block aggregation have been developed in the literature, principally by Askari-Nasab et al. (2010); Tabesh and Askari-Nasab (2011); Tabesh and Askari-Nasab (2013); and Badiozamani and Askari-Nasab (2014) to deal with uncertainties in short-term planning and to create mining polygons that can be used as planning units in the next planning stages ( Tabesh and Askari-Nasab 2019). Similar bloc units are grouped into a similarity index with the control of the shape and size of the polygons.

Two clustering models can be used: hierarchical clustering, partitional clustering, and k-means clustering. K-Means consists of choosing the number K of clusters before selecting at random K points and the centroids from the datasets. Each data point was then assigned to the nearest point to create K clusters around the centroid. This process consists of computing and placing the new centroid of each cluster. Each data point was assigned to the new nearest centroid if the outcome was unsatisfactory. If any reassignment occurred, we had to compute again to obtain a good result. Compared to hierarchical clustering, the K-means clustering method is reported to be faster but less accurate ( Feng et al. 2010).

Hierarchical clustering is categorized in two types: agglomerative and divisive.

Agglomerative is a bottom-up approach useful for mining planning that uses the similarity of an object to merge to form one object. First, each data point is made into a single-point cluster to form N clusters. The two closest data points are merged to form one cluster using Euclidean distance. The number of similar clusters will be reduced from N to N1 clusters. The algorithm will continue to have at the end one huge cluster grouping similar objects.

Divisive algorithms are the reverse of the agglomerative algorithms. This divides the units of a cluster into two smaller groups. Consequently, the divisive algorithms stop when the number of clusters is equal to the number of objects. The common concepts between the two groups are the definition of similarity measures and ways to update the similarity values when new clusters are defined ( Tabesh and Askari-Nasab 2013).

Salman et al. (2021) used the K-means clustering algorithm with a heuristic approach to obtain mineable shapes using the Newman and Marvin datasets related to the copper deposit ( Newman et al. 2010). This algorithm is applicable on a bench-by-bench basis and for metallic deposits.

Material destination homogeneity and mining direction constraints were applied to obtain minable cluster shapes. The objective was to reduce the costs of optimizing short-term mine planning. In contrast to the hierarchical clustering of Tabesh and Askari-Nasab (2019), this algorithm does not consider multiple elements with grade uncertainty.

Table 3 presents the results of different techniques for mining polygons using and completing the table of Sari and Kumral (2018).

## 4. Review of Short-Term mine Planning Optimization methods for open pits Mine

According to published research, the optimization of short-term mine planning is based on the objective functions, constraints, and level of modelling of the specifics of mining operations. The objective is to minimize the operating costs, minimize the deviation present in the quantity and quality of the produced ore from the LOM planning ( Jewbali and Dimitrakopoulos 2018), and maximize the utilization of available equipment ( Matamoros and Dimitrakopoulos 2016; Osanloo and Rahmanpour 2017; Upadhyay and Nasab 2017).

Mathematical programming algorithms (linear, nonlinear, and mixed integer programming), metaheuristics, stochastic simulations, and simulation annealing approaches have been applied for short-term mining planning optimization.

**Figure 3.**
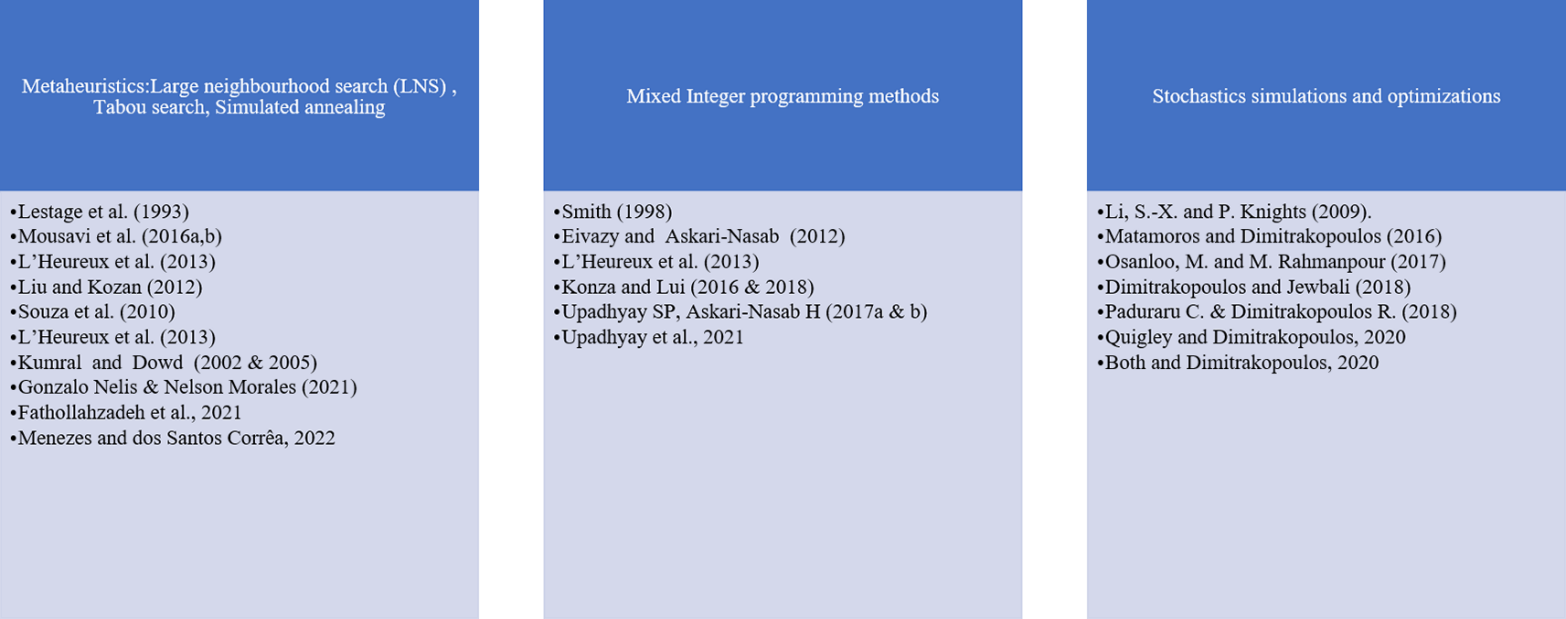
Short-term planning optimization method modified and updated from Blom et al. (2019).

Figure 3 presents the main approaches used for short-term planning optimization.

### 4.1 Mixed Integer programming (MIP) methods

Mixed Integer programming (MIP) is a mathematical optimization in which the objective function and constraints are linear or non-linear, and variables are integer, discrete, and non-discrete in some cases.

The MIP model for mine planning was developed by Gershon (1982) and Gershon (1983) to maximize NPV with blending and processing constraints. This optimization model can also minimize cost, maximize production, and maximize NPV. In contrary to the stochastic optimization, Eivazy and Askari-Nasab (2012) mentioned this method doesn’t capture the cost uncertainty related to the change of input data (Geological Bloc model, cost, prices, recoveries and mining constraints).

The advantage of MIP in short-term planning is the limitation of the size problem to be solved, which requires close attention to a number of algorithmic parameters and solutions ( Smith 1998), because fewer blocks are considered in problems than in long-term planning. Only the blocks scheduled to be extracted within the specified time horizon may be considered in the MIP model of the short-term planning problem ( L’Heureux et al. 2013).

Depending on the mining operations to be modelled and the required level of detail for the planning, MIP programming has been widely used to perform short-term planning in the last decade ( L’Heureux et al. 2013; Upadhyay and Nasab 2017; Upadhyay and Askari-Nasab 2018; Blom et al. 2019) MIP methods need the necessary decision variables and indices at time t to represent each mining operation (drilling and blasting, loading, hauling, stockpiling, etc.) to be modelled and included in the schedule for each block or block set of the geological model.

Eivazy and Askari-Nasab (2012) developed an MIP model to minimize mining, hauling, stockpiling, rehandling, processing, and waste rehabilitation costs for short-term mine planning of an iron mine on a monthly basis to track long-term mining planning. He used a branch-and-cut algorithm using the TOMLAB/CPLEX optimizer to solve this problem by considering some constraints (precedence, mining and processing capacities, grade processing requirements) and multiple destinations and mining directions.

L’Heureux et al. (2013) used MIP in short-term mine planning to address the issue of shovel movement and production capacity of equipment on a daily to monthly basis. They used the CPLEX optimization method. The objective is to minimize the cost of mining operations such as drilling, blasting, and extraction, respecting the precedence of these activities to constrain shovel movements. Shovels were assigned to a face (block or set of blocs) for the time horizon. Variable decisions were used for each period for shovel-to-face assignments, face-to-face movement of shovels, and drilling, blasting, and extraction decisions.

Kozan and Liu (2016) and Kozan and Liu (2018) proposed a multi-stage mine production scheduling (MPS) to optimize multi-resource multi-stage timetables at the operational level to determine how and when the equipment will be allocated to the selected blocks (or set of blocks) to perform mining operations (drilling, blasting, and extraction) over a short period of time under equipment capacity constraints for iron mines on a weekly basis. Their MIP model was solved by CPLEX optimization to minimize the mining operation cost (minimizing all of the equipment’s idle times at each stage). and maximize mining productivity and utilization of mining equipment through multiple processing stages.

Upadhyay and Askari-Nasab (2018) proposed a Mixed Integer Linear Goal Programming (MILGP) model solved by CPLEX, which provides shovel allocation decisions based on available faces to generate short-term production schedules capturing operational uncertainty. The integer variable represents the number of truck trips between the faces and destination. The objective is to maximize production, optimize ore tonnage received at processing plants, minimize deviations in grade blending requirements from various ore destinations, and minimize shovel movement. Later, ( Upadhyay et al. 2021) improved their model to be more practical by allocating shovels continuously on mining cuts.

Sari and Kumral (2018) used mixed-integer linear programming (MILP) to solve the dig-limits between ore and waste problems for short-term open-pit mine operations that are compatible with the maneuvering capability of the excavator and minimize dilution/loss, as opposed to free selection of ore and waste SMUs based on the cut-off grade. The objective is to maximize profit with block aggregation of ore or waste.

In five-week time horizons, Nelis and Morales (2022) used MIP optimization to define mineable mining cuts and mining sequences for a copper mine. The objective is to perform mining cuts and dig-limits by considering the cutoff grade, mining precedence (representative of SMU), and defining the destination of materials in each bench to produce a better evaluation of real NPV in the long term. The mining polygons complied with operational constraints such as the size of the loading equipment.

### 4.2 Stochastics optimization methods

Stochastic optimization has recently been developed for short-term planning ( Matamoros and Dimitrakopoulos 2016; Jewbali and Dimitrakopoulos 2018). It has been relatively well developed for long-term planning over the last decade and is one of the best methods for mine planning optimization. This integrates the parameter uncertainties used in the plan. In contrast to deterministic programming, which applies a constant parameter, stochastic programming uses a probabilistic approach that uses a probability distribution of each parameter to be applied to each set of possible realizations.

Mining parameters (grades, operating costs, commodity prices, recoveries) and the operational constraints estimated on the data at the time of planning are considered risky assets because of their fluctuation during the execution of this plan ( Osanloo and Rahmanpour 2017).

The goal of stochastic programming for short-term mine planning is to minimize this risk or maximize the predicted objective value of the decision variables. Two decision variable stages can be defined according ( Blom et al. 2019), the first-stage decision variables define the plan to be executed, and the variables for the second stage are determined by each potential scenario and indicate the changes that would need to be made to the plan if the scenario came to pass.

LI and KNIGHTS (2009) have advised the application of real option pricing theory from financial uncertainty and risk as stochastic optimization for medium- and short-term planning, taking into account fuel price fluctuations. To minimize operating costs and overcome this variation, the authors proved a significant economic benefit by incorporating stochastic analysis in truck dispatching. The objective is to ensure that the trucks dump waste to the closest waste dump during the period of high fuel price to reduce the number of trucks used. In periods of low fuel prices, waste is hauled directly to the under-filled dumps with the target to balance the storage volume across the set of available dumps.

As uncertainties cause deviation from the long-term plan, Matamoros and Dimitrakopoulos (2016) presented stochastic integer programming for a short-term mining plan for the iron open-pit mine case. The first stage is to minimize mining costs (loading, hauling, and availability of faces and equipment) and maximize fleet utilization. The second stage is to minimize the cost over the range of recourse costs associated with deviations from the target plan in terms of geology (ore tons, grade, and deleterious elements) and fleet (mechanical availability and hauling time) uncertainties. The authors demonstrated that the solution of this stochastic formulation is better than a deterministic plan with a lower cost because it takes into account the fluctuations of the ore quality and fleet parameters. This model was improved by Quigley and Dimitrakopoulos (2020) by generating a short-term plan that minimizes the shovel movement and production deviation (ton and grade sent to the plant allocated). This model has been applied to copper mines.

Contrary to the deterministic approach of mining planning, which consists of scheduling production first and then allocating fleets, Both and Dimitrakopoulos (2020), have developed a new model for stochastic optimization by using a metaheuristic solution to solve the problem that simultaneously optimizes the short-term extraction sequence and fleet management. The model considers geological, equipment performance, and truck cycle–time uncertainties.

Combining Fuzzy linear programming (non-linear programming) and optimization of risky assets Osanloo and Rahmanpour (2017) has applied portfolio optimization to short-term planning to minimize mining costs and risky assets, and to maximize the expected return of the portfolio by investing a particular amount of money. For authors, the uncertainties come from the capacity of mining faces to comply with the plant requirements (tonnage and grade) after material blending (portfolio) of different faces, taking into account their geological characteristics (return). In relation to the limestone mine, the extraction capacity of each location was defined, and each mining face was considered as the extent of investment for each asset. All parameters are bounded numbers and are fuzzified (equipment capacities, grades, recoveries, etc.).

Paduraru and Dimitrakopoulos (2018) proposed state-dependent policies to help mine complexes respond to new information (grade control results) for adapting short-term mining plans for material-feeding plants. The authors applied this model to gold and copper mines with six possible destinations. The objective is to assist and make the best decision to reassign the update blocks mined estimate to the new destination, to improve revenue, and to minimize the processing cost via stockpiling management.

The last work with stochastic planning was carried out by Jewbali and Dimitrakopoulos (2018) by combining long- and short-term planning through simulated grade control data to obtain a new estimate of ore body unavailable at the time of schedule setup. To reduce the deviation from long-term planning and satisfy the production plan, the authors proposed a multi-stage approach to the planning process by generating possible data for future grade control plans. Stochastic integer programming was applied to simulate the new ore body estimate via the grade control data generated. The objective was to maximize the NPV in long-term planning and minimize the cost of the production target within the long-term plan.

### 4.3 Metaheuristic and Heuristic Methods

Metaheuristics is a mathematical optimization technique with the goal of efficiently exploring the search space and finding near-optimal solutions. However, there is no guarantee of feasibility and optimality of the resulting solutions, although it provides good near-optimal solutions within a reasonable time. Fathollahzadeh et al. (2021) classified heuristic and metaheuristic methods. There are a wide variety of metaheuristic approaches, and most solutions used in short-term mining planning are as follows:

• Tabu Search (TS),
• simulated annealing approach (SA)
• Large neighbourhood search (LNS)
• Ant colony optimisation (ACO)

The Tabu Search (TS) is a metaheuristic method for mathematical optimization that is used for local or neighborhood searches and it has been by Glover (1986).

This optimization method iteratively moves from one potential solution to a better solution in the same neighborhood space. As the search progresses, Tabu search carefully explores the neighborhood of each solution and saves the results in a list of potential solutions to consider. If a potential solution appears in the tabu list, it cannot be revisited until it expires. The size of the tabu list should be large enough to prevent cycling but not so large that it prohibits too many moves. Liu and Ong (2004) and TS ability to prevent visiting previously recorded solutions ( Mousavi et al. 2016a).

Simulate annealing (SA) algorithm was inspired by the analogy of thermodynamics, in which metals are cooled and annealed ( Kirkpatrick et al. 1983). It is a metaheuristic approach known for finding a better solution when one is obstructed by a non-improving solution, and it can be viewed as a special form of TS, where a move becomes a tabu with a specified probability.

Shaw (1998) applied a large neighborhood search (LNS) has been applied by Shaw (1998). Unlike common neighborhood search, LNS uses a destroy-and-repair mechanism to define a new solution. According to this mechanism, part of the solution is destroyed, and then a repair technique rebuilds the solution ( Pisinger and Ropke 2010). For mine planning, this method involves moving blocks between periods and destinations.

Few studies have described metaheuristic problems in short-term mining planning. Shishvan and Sattarvand 2015 developed an Ant Colony Optimization (ACO) metaheuristic to solve an extended MBS-type problem applied to a copper–gold mine for long-term mining planning.

One of the last works for a metaheuristic approach for short-term planning was proposed by Mousavi et al. (2016a) comparing metaheuristic algorithms: Tabu Search (TS), simulated annealing (SA), and a hybrid solution SA and TS to solve the Open Pit Blocks Sequencing Problem (OPBS). These methods include mining constraints, stockpiling, ore blending, machine workspace to extract a given block from four possible sides, and dynamic destination assignment on a daily or weekly basis. The objective of the proposed OPBS model is to minimize stockpiling costs, including rehandling, holding costs, and satisfying processing requirements. To improve the TS and SA solutions, the authors proposed a hybrid TS-SA algorithm where SA embedded in the TS framework is used to accept some non-improving moves to allow a more diversified investigation of the solution space.

Mousavi et al. (2016a) proposed another hybrid metaheuristic short-term planning optimization method. To solve the Model Block Sequencing (MBS) problem, they hybridized the simulated annealing (SA), large neighborhood search (LNS), and branch-and-bound (BB) approaches used by Eivazy and Askari-Nasab (2012). A partial neighborhood solution (PNS) is constructed in each iteration of the SA. Then, a full. neighbourhood solution (NS) is achieved by assigning destinations to blocks using the B&B algorithm. The objective is to minimize stockpiling costs, including rehandling, holding costs, and satisfying processing requirements.

Liu and Kozan (2012) proposed a hybrid shifting bottleneck procedure (HSBP) algorithm combined with the Tabu Search (TS) metaheuristic algorithm has been developed to deal with the parallel-machine job-shop scheduling (PMJSS) problem. The objective is to improve the existing solutions to a short-term planning problem with equipment assignment.

Kumral and Dowd (2005) proposed a simulated annealing metaheuristic combined with Lagrangian relaxation and a multi-objective simulated annealing model (MOSA) to determine the optimal short-term production to reach an optimal or near-optimal schedule.

Similar to metaheuristic methods, Menezes and dos Santos Corrêa (2022) proposed a heuristic method, a new integrated mathematical method that considers quality, metallurgical recovery, mass balance, and stockpiling for an iron mine. The objective is to minimize operating costs by using a set of algorithms to manage the variations in production capacity for mining supply chains and meeting customer demands. To find the optimal solution for short-term mining planning, the authors used heuristic algorithms (relax, fix, fix, and optimize) and mixed-integer programming (local branching) using CPLEX.

## 5. Discussion and Conclusions

This paper reviews the literature for short-term mine planning optimization in order to address the main methods to perform the mine polygon basis elements of short-term planning as well as the main mathematical models and solution techniques for short-term mine planning optimization. To avoid manual mining cuts, clustering and dig-limit optimization are two useful methods for automatically mining cuts and optimizing short-term mine plans ( Tabesh and Askari-Nasab 2013; Tabesh and Askari-Nasab 2019; Salman et al. 2021; Nelis et al. 2022). To maximize profit, the integration of cut-off grade in mining polygons is very important to define each material with its destination.

However, in addition to the non-clarification of the optimal number of clusters to be determined ( Vasylchuk and Deutsch 2019), clustering can imply some loss of information by merging blocks that can affect the NPV of the mining operation in the strategic plan ( Salman et al. 2021) even if it can satisfy the short-term plan requirement.

The dig-limit optimization method could be the best alternative to the clustering method for mining polygons. For this, a geological block model must be prepared with a good grade control program to reduce uncertainties and risks in the delimitation of ore bodies.

The key to success in short-term mine planning is to model all operations (drilling, blasting, loading, hauling, stockpiling, materials rehandling, and processing) in detail to identify bottlenecks in each operation and to facilitate the full reconciliation between long-term and short-term mining planning. The daily or weekly basis of short-term mine planning is to have a good assignment of shovels and trucks for Ore and Waste for the different destinations to meet production targets and ensure the best quality of ore to the process requirement aligned to the long-term mine planning.

Owing to their good ability, metaheuristics provide a useful platform for global optimization to tackle large-scale nonlinear optimization models in a reasonable time ( Goodfellow and Dimitrakopoulos 2016). Many authors have used a hybrid method, mixed integer programming, and metaheuristics methods ( Liu and Kozan 2012; Mousavi et al. 2016b; Mousavi et al. 2016a; Fathollahzadeh et al. 2021) and also included uncertainties with stochastic optimization to improve the mining sequence in the short-term mine plan ( Matamoros and Dimitrakopoulos 2016; Jewbali and Dimitrakopoulos 2018) because using each method separately limits the results of short-term mining plans. Habib et al. (2023) presented since 2010 stochastic comparison in recent studies. It is clear that short-term mining planning remains a challenge for stochastic areas.

In the current state-of-the-art commercial tools and software (Datamine, Deswik, Vulcan), it is difficult to resolve short-term mine planning optimization according to their level of development and capacity in a number of blocks. Stochastic optimization for short-term mine plans is starting to be developed in some cases. According to our literature review, many authors use CPLEX, Python, Arena, or UDESS as an alternative to optimize planning.

This literature review is a good basis for moving forward to the best optimization method for our next topic research dedicated to short-term mining plan optimization.

**Figure 4.**
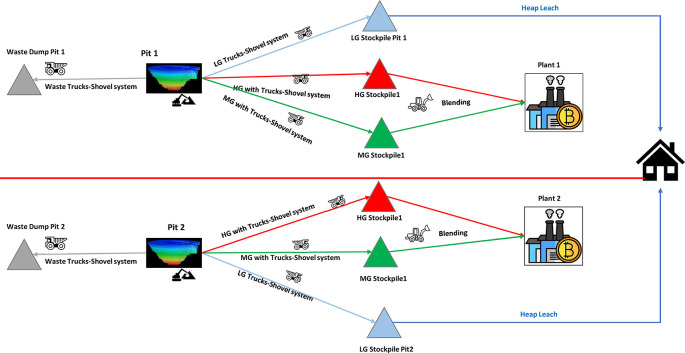
Flowchart for mining operations Mining operations with TS from pit to stockpiles, plants and waste dumps.

The subject will try to implement a short-term mining plan to align with the long-term goals of gold open-pit mine operations, as shown in Figure 4.

## Ethics and consent

Ethical approval and consent were not required.

## Data Availability

No data are associated with this article.

## References

[ref1] Askari-Nasab H Tabesh M Badiozamani MM : Creating mining-cuts using hierarchical clustering and tabu search algorithms. *Min. Optim. Lab.* 22;2010.

[ref2] Badiozamani MM Askari-Nasab H : Integration of reclamation and tailings management in oil sands surface mine planning. *Environ. Model Softw.* 2014;51:45–58. 10.1016/j.envsoft.2013.09.026

[ref3] Blom M Pearce AR Stuckey PJ : Short-term planning for open pit mines: a review. *Int. J. Min. Reclam. Environ.* 2019;33(5):318–339. 10.1080/17480930.2018.1448248

[ref4] Both C Dimitrakopoulos R : Joint stochastic short-term production scheduling and fleet management optimization for mining complexes. *Optim. Eng.* 2020;21(4):1717–1743. 10.1007/s11081-020-09495-x

[ref5] Caccetta L Hill SP : An application of branch and cut to open pit mine scheduling. *J. Glob. Optim.* 2003;27(2–3):349–365. 10.1023/A:1024835022186

[ref6] Chanda EK Wilke FL : An EPD model of open pit short term production scheduling optimization for stratiform orebodies. *Proc 23rd APCOM Symp. [place unknown].* 1992; pp.759–768.

[ref7] Eivazy H Askari-Nasab H : A mixed integer linear programming model for short-term open pit mine production scheduling. *Trans. Institutions Min. Metall. Sect. A Min. Technol.* 2012;121(2):97–108. 10.1179/1743286312Y.0000000006

[ref8] Fathollahzadeh K Asad MWA Mardaneh E : Review of Solution Methodologies for Open Pit Mine Production Scheduling Problem. *Int. J. Min. Reclam. Environ.* 2021;35(8):564–599. 10.1080/17480930.2021.1888395

[ref9] Feng L Qiu MH Wang YX : A fast divisive clustering algorithm using an improved discrete particle swarm optimizer. *Pattern. Recogn. Lett.* 2010;31(11):1216–1225. 10.1016/j.patrec.2010.04.001

[ref10] Gershon M : Linear Programming Approach To Mine Scheduling Optimization. *Appl. Comput. Oper. Res. Miner. Ind. [place unknown].* 1982; pp.483–493.

[ref11] Gershon ME : Mine Scheduling Optimization With Mixed Integer Programming. *Min. Eng.* 1983;35(4):351–354.

[ref12] Glover F : Future paths for integer programming and links to artificial intelligence. *Comput. Oper. Res.* 1986;13(5):533–549. 10.1016/0305-0548(86)90048-1

[ref13] Goodfellow RC Dimitrakopoulos R : Global optimization of open pit mining complexes with uncertainty. *Appl. Soft. Comput. J.* 2016;40:292–304. 10.1016/j.asoc.2015.11.038

[ref14] Habib N Al Ben-awuah E Askari-nasab H : Review of recent developments in short-term mine planning and IPCC with a research agenda. *Min. Technol.* 2023;132:179–201. 10.1080/25726668.2023.2218170

[ref15] Isaaks E Treloar I Elenbaas T : Optimum dig lines for open pit grade control. *Proc. 9th Int. Min. Geol. Conf. [place unknown].* 2014; pp.425–432.

[ref16] Jewbali A Dimitrakopoulos R : Stochastic mine planning-example and value from integrating long-and short-term mine planning through simulated grade control, Sunrise Dam, Western Australia. *Adv Appl Strateg Mine Plan. [place unknown].* Springer;2018; pp.173–190. 10.1007/978-3-319-69320-0_13

[ref17] Juarez G Dodds R Echeverría A : Open pit strategic mine planning with automatic phase generation. *OREBODY Model Strateg MINE Plan Symp. Proceedings. AusIMM, Perth. [place unknown].* 2014; pp.24–26.

[ref18] Kahraman MM Dessureault SD : Development of a real-time adherence to mine plan tools as part of an integrated remote mine control centre. *35th Int. Symp. Appl. Comput. Oper. Res. Miner. Ind. APCOM. [place unknown].* 2011; pp.819–825.

[ref19] King B : Modelling Open Pit and Underground Production Scheduling in Concert. *Orebody Model Strateg. Mine. Plan 2014. [place unknown].* 2014; pp.369–373.

[ref20] Kirkpatrick S Gelatt CD Vecchi MP : Optimization by simulated annealing. *Science (80-).* 1983;220(4598):671–680. 10.1126/science.220.4598.671 17813860

[ref21] Kozan E Liu SQ : Operations research for mining: A classification and literature review. *ASOR Bull.* 2011;30(1):2–23.

[ref22] Kozan E Liu SQ : A new open-pit multi-stage mine production timetabling model for drilling, blasting and excavating operations. *Trans. Institutions Min. Metall. Sect. A Min. Technol.* 2016;125(1):47–53. 10.1179/1743286315Y.0000000031

[ref23] Kozan E Liu SQ : An open-pit multi-stage mine production scheduling model for drilling, blasting and excavating operations. *Adv. Appl. Strateg. Mine. Plan.* 2018;655–668. 10.1007/978-3-319-69320-0_38

[ref24] Kumral M Dowd PA : A simulated annealing approach to mine production scheduling. *J. Oper. Res. Soc.* 2005;56(8):922–930. 10.1057/palgrave.jors.2601902

[ref25] L’Heureux G Gamache M Soumis F : Mixed integer programming model for short term planning in open-pit mines. *Trans. Institutions Min. Metall. Sect. A Min. Technol.* 2013;122(2):101–109. 10.1179/1743286313Y.0000000037

[ref26] Lamghari A : Mine Planning and Oil Field Development: A Survey and Research Potentials. *Math. Geosci.* 2017;49(3):395–437. 10.1007/s11004-017-9676-z

[ref27] Li SX Knights P : Integration of real options into short-term mine planning and production scheduling. *Min. Sci. Technol.* 2009;19(5):674–678. 10.1016/S1674-5264(09)60125-3

[ref28] Liu SQ Kozan E : A hybrid shifting bottleneck procedure algorithm for the parallel-machine job-shop scheduling problem. *J. Oper. Res. Soc.* 2012;63(2):168–182. 10.1057/jors.2011.4

[ref29] Liu SQ Ong HL : Metaheuristics for the mixed shop scheduling problem. *Asia-Pacific J. Oper. Res.* 2004;21(1):97–115. 10.1142/S0217595904000072

[ref30] Marques R d S Souza LE de Albarnaz LD : Modeling and planning of bentonite clay mining: a case study at Bañado de Medina, Melo, Uruguay. *Rem. Rev. Esc. Minas.* 2013;66(4):521–528. 10.1590/s0370-44672013000400018

[ref31] Matamoros MEV Dimitrakopoulos R : Stochastic short-term mine production schedule accounting for fleet allocation, operational considerations and blending restrictions. *Eur. J. Oper. Res.* 2016;255(3):911–921. 10.1016/j.ejor.2016.05.050

[ref32] Menezes GC Santos Corrêa J dos : Model and algorithms applied to Short-Term Integrated Programming Problem in Mines. *Res. Policy.* 2022;79(January):102950. 10.1016/j.resourpol.2022.102950

[ref33] Mohtasham M Mirzaei-Nasirabad H Askari-Nasab H : Truck fleet size selection in open-pit mines based on the match factor using a MINLP model. *Min. Technol.: Trans. Inst. Min. Metall.* 2021;130(3):1–17. 10.1080/25726668.2021.1919374

[ref34] Moradi Afrapoli A Askari-Nasab H : Mining fleet management systems: a review of models and algorithms. *Int. J. Min. Reclam. Environ.* 2019;33(1):42–60. 10.1080/17480930.2017.1336607

[ref35] Mousavi A Kozan E Liu SQ : Comparative analysis of three metaheuristics for short-term open pit block sequencing. *J. Heuristics.* 2016a;22(3):301–329. 10.1007/s10732-016-9311-z

[ref37] Mousavi A Kozan E Liu SQ : Open-pit block sequencing optimization: A mathematical model and solution technique. *Eng. Optim.* 2016b;48(11):1932–1950. 10.1080/0305215X.2016.1142080

[ref38] Nelis G Meunier F Morales N : Column Generation for Mining Cut Definition with Geometallurgical Interactions. *Nat. Resour. Res.* 2022;31(1):131–148. 10.1007/s11053-021-09976-5

[ref39] Nelis G Morales N : A mathematical model for the scheduling and definition of mining cuts in short-term mine planning. *Optim. Eng.* 2022;23(1):233–257. 10.1007/s11081-020-09580-1

[ref40] Neufeld CT Norrena KP Deutsch CV : Semi-Automatic Dig Limit Generation. *Cent Comput Geostatistics Annu Rep Pap. [place unknown].* 2003; pp.1–23. papers2://publication/uuid/5155E453-EB0A-4808-9452-B86CAA180583.

[ref41] Newman AM Rubio E Caro R : A review of operations research in mine planning. *Interfaces (Providence).* 2010;40(3):222–245. 10.1287/inte.1090.0492

[ref42] Noriega R Pourrahimian Y : A systematic review of artificial intelligence and data-driven approaches in strategic open-pit mine planning. *Res. Policy.* 2022;77(November 2021):102727. 10.1016/j.resourpol.2022.102727

[ref43] Norrena KP Deutsch CV : Automatic Determination of Dig Limits Subject to Geostatistical, Economic and Equipment Constraints. *Cent. Comput. Geostatistics Annu. Rep. Pap.* 2001;1–18. papers2://publication/uuid/8E3B6A0A-4131-4F32-8D36-406B0312C5C4.

[ref44] Osanloo M Gholamnejad J Karimi B : Long-term open pit mine production planning: A review of models and algorithms. *Int. J. Min. Reclam. Environ.* 2008;22(1):3–35. 10.1080/17480930601118947

[ref45] Osanloo M Rahmanpour M : Mining. 2017;70(1):109–116.

[ref46] Otto TJ Musingwini C : A compliance driver tree (CDT) based approach for improving the alignment of spatial and intertemporal execution with mine planning at open-pit mines. *Res. Policy.* 2020;69(February):101826. 10.1016/j.resourpol.2020.101826

[ref47] P NK Neufeld CT Deutsch CV : An Update on Automatic Dig Limit Determination. *Cent. Comput. Geostatistics Annu. Rep. Pap.* 2002;7:1–17. papers2://publication/uuid/FF1B87D4-7799-4EEB-837D-2067AF429CDC.

[ref48] Paduraru C Dimitrakopoulos R : Adaptive policies for short-term material flow optimization in a mining complex. *Min. Technol.: Trans. Inst. Min. Metall.* 2018;127(1):56–63. 10.1080/14749009.2017.1341142

[ref49] Pisinger D Ropke S : Large neighborhood search. *Handb metaheuristics. [place unknown].* Springer;2010; pp.399–419.

[ref50] Quigley M Dimitrakopoulos R : Incorporating geological and equipment performance uncertainty while optimising short-term mine production schedules. *Int. J. Min. Reclam. Environ.* 2020;34(5):362–383. 10.1080/17480930.2019.1658923

[ref51] Richmond A : Integrating multiple simulations and mining dilution in open pit optimisation algorithms. *Australas Inst. Min. Metall. Publ. Ser. [place unknown].* 2007; pp.317–322.

[ref52] Richmond AJ Beasley JE : Financially efficient dig-line delineation incorporating equipment constraints and grade uncertainty. *Int. J. Surf. Min. Reclam. Environ.* 2004;18(2):99–121. 10.1080/13895260412331295376

[ref53] Ruiseco JR Williams J Kumral M : Optimizing Ore–Waste Dig-Limits as Part of Operational Mine Planning Through Genetic Algorithms. *Nat. Resour. Res.* 2016;25(4):473–485. 10.1007/s11053-016-9296-1

[ref54] Salman S Muhammad K Khan A : A Block Aggregation Method for Short-Term Planning of Open Pit Mining with Multiple Processing Destinations. *Minerals.* 2021;11(3):288. 10.3390/min11030288

[ref55] Sari YA Kumral M : Dig-limits optimization through mixed-integer linear programming in open-pit mines. *J. Oper. Res. Soc.* 2018;69(2):171–182. 10.1057/s41274-017-0201-z

[ref57] Shaw P : Using constraint programming and local search methods to solve vehicle routing problems. *Lect. Notes Comput. Sci. (including Subser Lect. Notes Artif. Intell. Lect. Notes Bioinformatics). Vol. 1520. [place unknown].* 1998; pp.417–431. 10.1007/3-540-49481-2_30

[ref58] Shishvan MS Sattarvand J : Long term production planning of open pit mines by ant colony optimization. *Eur. J. Oper. Res.* 2015;240(3):825–836. 10.1016/j.ejor.2014.07.040

[ref59] Smith ML : Optimizing short-term production schedules in surface mining: Integrating mine modeling software with AMPL/CPLEX. *Int. J. Surf. Min. Reclam. Environ.* 1998;12(4):149–155. 10.1080/09208118908944038

[ref60] Tabesh M Askari-Nasab H : Two-stage clustering algorithm for block aggregation in open pit mines. *Trans. Institutions Min. Metall. Sect. A Min. Technol.* 2011;120(3):158–169. 10.1179/1743286311Y.0000000009

[ref61] Tabesh M Askari-Nasab H : Automatic creation of mining polygons using hierarchical clustering techniques. *J. Min. Sci.* 2013;49(3):426–440. 10.1134/S1062739149030106

[ref62] Tabesh M Askari-Nasab H : Clustering mining blocks in presence of geological uncertainty. *Min. Technol.: Trans. Inst. Min. Metall.* 2019;128(3):162–176. 10.1080/25726668.2019.1596425

[ref63] Upadhyay S Nasab HA : Simulation and Optimization of Mine Operations for Desired Grade Blending in Open Pit Mines. *APCOM. [place unknown].* 2017; pp.1–11.

[ref64] Upadhyay SP Askari-Nasab H : Simulation and optimization approach for uncertainty-based short-term planning in open pit mines. *Int. J. Min. Sci. Technol.* 2018;28(2):153–166. 10.1016/j.ijmst.2017.12.003

[ref66] Upadhyay SP Askari-Nasab H Tabesh M : Simulation and optimization in open pit mining. *Proc. Appl. Comput. Oper. Res. Miner. Ind. 37th Int. Symp. APCOM. [place unknown].* 2015.

[ref67] Upadhyay SP Doucette J Askari-Nasab H : Short-term production scheduling in open pit mines with shovel allocations over continuous time frames. *Int. J. Min. Miner. Process. Eng.* 2021;12(4):292–308. 10.1504/IJMME.2021.121325

[ref68] Vasylchuk YV Deutsch CV : Optimization of Surface Mining Dig Limits with a Practical Heuristic Algorithm. *Mining, Metall. Explor.* 2019;36(4):773–784. 10.1007/s42461-019-0072-8

[ref69] Wilde BJ Deutsch CV : A Short Note Comparing Feasibility Grade Control with Dig Limit Grade Control. *Cent. Comput. Geostatistics.* 2007;1–10.

