# Supplementary material for: State-of-the-art optimization methods for short-term mine planning
Source: F1000Res. 2025 Apr 15;13:1107. Originally published 2024 Sep 30. [Version 2] doi: 10.12688/f1000research.152986.2 (PMC12498321; doi:10.12688/f1000research.152986.2)
Supplement: Peer review history

# Reviewer report: Reviewer response for version 2

**DOI:** 10.5256/f1000research.177698.r379288

**Published:** 2025-05-21

**Authors:** Francis F Pavloudakis

**Affiliations:** 1 University of Western Macedonia, Kozani, Greece

Dear authors,

 Your article is quite interesting and addresses an issue that concerns every mining operation. Overall, the article discusses methods and techniques for optimizing short-term mine planning. However, I believe some areas require improvement.

1. Introduction: In the text, you mention "quite a few" reviews on long-term planning and "very few" for short-term planning. However, Figure 1 indicates the opposite. Ultimately, is "monthly to yearly" time discretization considered long-term or short-term planning? Also, where exactly does medium-term planning fit in? If the plan duration is not the decisive factor for distinguishing short-term from long-term planning, please clearly explain what that factor is in your opinion.
2. Introduction / Section 1.1: You mention certain available software while omitting others (additional software is mentioned in a later section).
3. Introduction / Section 1.1: "algorithms have been are applied..." Please correct this sentence.
4. Figure 1: The font size of the embedded diagrams is small.
5. Tables: Please maintain consistent font type and size throughout.
6. Scope of the research: Please add specific details on how you searched for the articles included in your review (e.g., keywords used in search engines, exclusion criteria for specific articles).
7. Sections 3 and 4: In my opinion, the content of these sections should be enriched with more information so that your article becomes useful even to a mining engineer who has not previously dealt with improving short-term mine planning. In other words, more details on methods and techniques should be provided rather than simply listing case studies conducted by researchers worldwide. For better organization and presentation of this information, I believe you can start by revisiting the Discussion and Conclusions section, which is the most well-written and clear in the entire article.

Overall, your article holds promise, and addressing these points could significantly enhance its clarity and impact.

 Best regards

Is the review written in accessible language?

Yes

Are all factual statements correct and adequately supported by citations?

Yes

Are the conclusions drawn appropriate in the context of the current research literature?

Yes

Is the topic of the review discussed comprehensively in the context of the current literature?

Yes

Reviewer Expertise:

Surface mining, Coal, Energy, Environment

I confirm that I have read this submission and believe that I have an appropriate level of expertise to confirm that it is of an acceptable scientific standard, however I have significant reservations, as outlined above.

# Reviewer report: Reviewer response for version 2

**DOI:** 10.5256/f1000research.177698.r378064

**Published:** 2025-05-21

**Authors:** Andrea Brickey

**Affiliations:** 1 Mining Engineering and Management Department, South Dakota School of Mines and Technology, Rapid City, South Dakota, USA

The authors have made improvements to the article and that work is appreciated.  However, there are still considerations and corrections that should be addressed before finalizing.  

 1. The title has not been changed despite the response to review 1 indicating that the new title would include "open-pit".

 2. Suggest changing to "under the guidance of the LOM." 1.1 - Short-term mine planning deals with the equipment and resources allocated on a monthly, weekly, daily, or shift-by-shift basis under the control of the LOM ( Noriega and Pourrahimian 2022).

 3. The authors use the term granularity, but it is unclear as to if the author is discussion the time horizon or time fidelity, two different aspects of planning.

 4. "To ensure the link...." this sentence has medium-term capitalized while all references to short-term and long-term are not. 

 5. Last sentence before section 3 has geotechnical capitalized. 

 6. "Several techniques have been used to perform..."  Commas are incorrectly used in this sentence.

 7. Another paper that you might consider "Amoako et al. 2023"

 8.  There are still many randomly capitalized words.

 9. Define SMU.

 10. Figure 3 - text is quite small in comparison to the size of the figure.

 11. Section 4.2 - sentence "Combining Fuzzy linear programming..."  remove the word "has" before "applied portfolio optimization"

 12. Section 4.3 - random capitalization in bullets.

 13. Section 4.3 - "The Tabu Search..."  remove "and it has been"

 14. Paper could benefit from further grammatical review.

 15. Section 5 - "The dig-limit optimization method could be the best..."  This seems like opinion and lacks justification.

 16. Metaheuristics do not provide global optimization.  They can provide near-optimal solutions, but are not guaranteed to solve to optimality.

 17. Please review citations and complete missing information.

Is the review written in accessible language?

No

Are all factual statements correct and adequately supported by citations?

No

Are the conclusions drawn appropriate in the context of the current research literature?

Yes

Is the topic of the review discussed comprehensively in the context of the current literature?

Yes

Reviewer Expertise:

mining engineering, mine design, surface mine planning, underground mine planning, mine optimization, operations research, production scheduling, and reserve estimation.

I confirm that I have read this submission and believe that I have an appropriate level of expertise to confirm that it is of an acceptable scientific standard, however I have significant reservations, as outlined above.

# Reviewer report: Reviewer response for version 2

**DOI:** 10.5256/f1000research.177698.r378062

**Published:** 2025-04-30

**Authors:** Chengkai Fan

**Affiliations:** 1 Université Laval, Québec, Canada

The author addressed the problems in my comments. No more comments, GREAT WORK.

Is the review written in accessible language?

Yes

Are all factual statements correct and adequately supported by citations?

Yes

Are the conclusions drawn appropriate in the context of the current research literature?

Yes

Is the topic of the review discussed comprehensively in the context of the current literature?

Partly

Reviewer Expertise:

Open pit mining, machine learning, mine transport, and rock mechanics.

I confirm that I have read this submission and believe that I have an appropriate level of expertise to confirm that it is of an acceptable scientific standard.

# Reviewer report: Reviewer response for version 1

**DOI:** 10.5256/f1000research.167816.r331462

**Published:** 2024-11-20

**Authors:** Pedro Henrique Alves Campos

**Affiliations:** 1 Federal University of Minas Gerais, Belo Horizonte,, MG, Brazil

The article is a review of short-term mine planning optimization methods. The authors focus on 1) optimizing dig limits(polygons), and 2) optimizing short-term production scheduling.

 I believe the paper has the potential to get indexed. However, some points need to be clarified and improved.

 01) The authors should highlight what this review presents as new concerning other reviews cited throughout the text, such as Blom et al. 2019, Fathollahzadeh et al. 2021, Al Habib el at. 2021 and Kozan et al. 2011.

 02) I suggest improving Table 1 by detailing the topics covered in each article review, their results, and main conclusions.

 03) There are several errors in writing, bad wording, comma misplacement, word repetition, etc. Examples:

 a) "...short-term mine planners with management teams are requested to identify and manage risks to mitigate them and adhere **able to stick** to the Life-of Mine (LOM) plan"

 b) "constraints to be **considered** and the level of details for mine operations to be **considered**"

 c) "similar **bloc** properties"

 d) "...(for example: Datamine RM,DatamineUG,SnowdenandNPV) **,H**owever,differentmodels and algorithms **,**are applied..."

 Therefore, I suggest doing a complete English review of the text.

 04) Tables 2 and 3 deserve to be more explained and carefully detailed (there is excessive use of ellipses)

 05) since we are already at the end of 2024, I believe the paper must be updated by referencing the latest papers on the topic. Also, the improvements and differences between the approaches of each research should be discussed more deeply.

 06) What is the importance of Figure 4 for your review? It does not seem to be relevant.

Is the review written in accessible language?

Partly

Are all factual statements correct and adequately supported by citations?

Partly

Are the conclusions drawn appropriate in the context of the current research literature?

Partly

Is the topic of the review discussed comprehensively in the context of the current literature?

Partly

Reviewer Expertise:

Geostatistics, mine planning, resource and reserves, machine learning

I confirm that I have read this submission and believe that I have an appropriate level of expertise to confirm that it is of an acceptable scientific standard, however I have significant reservations, as outlined above.

# Response

**Published:** 2025-01-15

**Authors:** Moise KAMBALA MALUNDAMENE

**Affiliations:** Mines, Ecole Nationale Superieure des Mines de Rabat, Agdal, Rabat-Sale-Zemmour-Zaer, Morocco

Hi Pedro,

 Happy new year,

 Thanks for your time to review our paper.

 Please, See below our responses to your comments

 1. The authors should highlight what this review presents as new concerning other reviews cited throughout the text, such as Blom et al. 2019, Fathollahzadeh et al. 2021, Al Habib el at. 2021 and Kozan et al. 2011.

 Response:

 Our sincere most gratitude to the reviewer for the valuable comments. In this review, we have added a dig limit review before proceeding with the short-term mine planning. We are also focusing on the daily to monthly planning while may review articles are based on monthly to yearly time discretisation,

 2. I suggest improving Table 1 by detailing the topics covered in each article review, their results, and main conclusions.

 Response:

 The last review article from (Habib et al. 2023), presented all these details. One of the focuses on this table is that few paper so far are modelling short-term in daily to monthly basis. Most of paper are using short-term from monthly to yearly as time discretisation. We have added one column to specify time discretization on each those review article of open pit mine.

 3. There are several errors in writing, bad wording, comma misplacement, word repetition, etc. Examples:

 a) "...short-term mine planners with management teams are requested to identify and manage risks to mitigate them and adhere able to stick to the Life-of Mine (LOM) plan"

 b) "constraints to be considered and the level of details for mine operations to be considered"

 c) "similar bloc properties"

 d) "...(for example: Datamine  

  RM,DatamineUG,SnowdenandNPV),However,different models and algorithms,are

 applied..."

 Therefore, I suggest doing a complete English review of the text.

 Response:

 The paper has been reviewed as suggested, thank you

 4. Tables 2 and 3 deserve to be more explained and carefully detailed (there is excessive use of ellipses)

 Response 

 The paper has been reviewed as suggested, thank you

 5. Since we are already at the end of 2024, I believe the paper must be updated by referencing the latest papers on the topic. Also, the improvements and differences between the approaches of each research should be discussed more deeply.

 Response:

 We have added some new references and this articles has been in process since the end of last year and following the process of the journal.

 6. What is the importance of Figure 4 for your review? It does not seem to be relevant.

 Response:

 The figure has been removed

 Thanks,

 Best Regards

 Moise

# Reviewer report: Reviewer response for version 1

**DOI:** 10.5256/f1000research.167816.r331461

**Published:** 2024-11-12

**Authors:** Andrea Brickey

**Affiliations:** 1 Mining Engineering and Management Department, South Dakota School of Mines and Technology, Rapid City, South Dakota, USA

This paper provides a review of the current state of short-term, open-pit, mine planning technical solutions.  The article provides a review of the challenges and documented solutions while highlighting areas where further technological improvements could be made. The paper is well cited with the current and relevant references. Yet, improvements should be made to increase clarity and quality.

 1. It is suggested that the authors add "surface" or "open-pit" to the title of the paper.

 2. In the abstract, the statement "short-term planning is more challenging because it deals with uncertainties: is vague and does not reflect the actual reality that all planning time horizons deal with uncertainties.

 3. There are numerous grammatical errors and typos throughout the document.  For example, mining planning is used in a few places.  The proper term would be mine planning.

 4. The introduction should make it clear that this paper is documenting the current state of open-pit or surface short-term mine planning.

 5. Review document the ensure that descriptions are using appropriate adjectives and not colloquial terms. 

 6. Inconsistent capitalization and formatting in numerous areas.  See Table 2 for some examples. 

 7. Some figures are too small to read, e.g., Figure 1.

 8. There are commercially available, heuristic-based scheduling solutions, e.g., RPM Global's XPAC.

 9. In some sections you refer to a geologic model as a "bloc model" and in other areas a "block model". I believe the industry standard is "block model".

 10.  Please make sure to use commas when listing more than 3 items.

 11. Define SMU in the text.

 12. It should be made clear that short-term solutions often determine destination. This is not explicitly stated in the paper.

 13. It seems that Figure 3 could be formatted to make the text larger.

 14. The statement on page 9 of the pdf file states, "They used the CPLEX optimization method." This should be stated as they used CPLEX to "solve" the formulation.

 15. Review document for proper tense.  See paragraph beginning with "Upadhyay and Askari-Nasab (2018)" on page 9 as an example.

 16. Section 4.2, first paragraph, should make it clear that these solutions are for "Strategic" mine planning. 

 17. The authors abbreviate branch-and-bound as BB, but then use the term as B&B. 

 18. Figure 4 does not seem to provide a significant amount of value to the paper.  It is suggested that either it be explained further in the document, simplified, or removed.

 19. On page 12, paragraph beginning with "Owing to their good ability..." the term "global optimization" seems to give the impression that metaheuristics provide optimal solutions.

 20. A few citations are included in this review for the authors' consideration. 

 Reference

 https://repository.mines.edu/bitstream/handle/11124/178655/Deutsch_mines_0052E_12734.pdf?sequence=2&isAllowed=y

Is the review written in accessible language?

No

Are all factual statements correct and adequately supported by citations?

No

Are the conclusions drawn appropriate in the context of the current research literature?

Yes

Is the topic of the review discussed comprehensively in the context of the current literature?

Yes

Reviewer Expertise:

mining engineering, mine design, surface mine planning, underground mine planning, mine optimization, operations research, production scheduling, and reserve estimation.

I confirm that I have read this submission and believe that I have an appropriate level of expertise to confirm that it is of an acceptable scientific standard, however I have significant reservations, as outlined above.

## References

[1] : MineLib: a library of open pit mining problems. *Annals of Operations Research* .2013;206(1) : 10.1007/s10479-012-1258-3 93-114 10.1007/s10479-012-1258-3

[2] : Production Scheduling for Strategic Open Pit Mine Planning: A Mixed-Integer Programming Approach. *Operations Research* .2020;68(5) : 10.1287/opre.2019.1965 1425-1444 10.1287/opre.2019.1965

# Response

**Published:** 2025-01-15

**Authors:** Moise KAMBALA MALUNDAMENE

**Affiliations:** Mines, Ecole Nationale Superieure des Mines de Rabat, Agdal, Rabat-Sale-Zemmour-Zaer, Morocco

Hello Andrea,

 Happy new year,

 Thanks for your time to review our paper.

 Please, See below our responses to your comments

 1. It is suggested that the authors add "surface" or "open-pit" to the title of the paper.

 Response:

 We thank the reviewer for the valuable insight to help us improve the content. The title has been updated to “State-of-the-art Optimization Methods of Open-Pit Short-Term Mine Planning” to reflect the focus on open-pit mining.

 2. In the abstract, the statement "short-term planning is more challenging because it deals with uncertainties" is vague and does not reflect the actual reality that all planning time horizons deal with uncertainties.

 Response:

 The sentence has been revised for clarity and context to: “Short-term planning deals with daily challenges, such as variations in block models and mining cut polygons.”

 3.There are numerous grammatical errors and typos throughout the document. For example, mining planning is used in a few places. The proper term would be mine planning.

 Response:

 The document has been reviewed thoroughly to correct grammatical errors and inconsistencies. The term “mine planning” has been used consistently throughout.

 4. The introduction should make it clear that this paper is documenting the current state of open-pit or surface short-term mine planning.

 Response:

 The introduction has been updated to explicitly state that the paper focuses on the current state of open-pit short-term mine planning.

 5. Review the document to ensure that descriptions are using appropriate adjectives and not colloquial terms.

 Response:

 Appropriate adjectives have been used, and colloquial terms have been removed to maintain a formal tone.

 6. Inconsistent capitalization and formatting in numerous areas. See Table 2 for some examples.

 Response:

 All capitalization and formatting inconsistencies have been corrected, including those in Table 2.

 7. Some figures are too small to read, e.g., Figure 1.

 Response:

 The text size in Figure 1 has been increased for better readability.

 8. There are commercially available, heuristic-based scheduling solutions, e.g., RPM Global's XPAC.

 Response:

 A discussion of heuristic-based scheduling solutions, including RPM Global’s XPAC, has been integrated into the conclusion.

 9. In some sections, you refer to a geologic model as a "bloc model" and in other areas as a "block model." I believe the industry standard is "block model."Response:

 The term “block model” has been consistently applied throughout the document.

 10. Please make sure to use commas when listing more than three items.

 Response:

 Commas have been correctly applied in lists containing more than three items.

 11. Define SMU in the text.

 Response:

 The term SMU (Small Mining Unit) has been defined in the text.

 12. It should be made clear that short-term solutions often determine destination. This is not explicitly stated in the paper.

 Response:

 This has been explicitly stated in Section 1.1, highlighting that short-term planning often determines material destinations.

 13. It seems that Figure 3 could be formatted to make the text larger.

 Response:

 The text in Figure 3 has been enlarged for clarity.

 14. The statement on page 9 of the PDF file states, "They used the CPLEX optimization method." This should be stated as they used CPLEX to "solve" the formulation.

 Response:

 The statement has been revised to indicate that CPLEX was used to “solve” the formulation.

 15.Review the document for proper tense. See paragraph beginning with "Upadhyay and Askari-Nasab (2018)" on page 9 as an example.

 Response:

 The grammar and tense in the document, including the mentioned paragraph, have been reviewed and corrected.

 16. Section 4.2, first paragraph, should make it clear that these solutions are for "Strategic" mine planning.

 Response:

 The first paragraph of Section 4.2 has been updated to clarify that the solutions discussed pertain to strategic mine planning.

 17. The authors abbreviate branch-and-bound as BB, but then use the term as B&B.

 Response:

 The abbreviation has been standardized as “B&B” throughout the paper.

 18. Figure 4 does not seem to provide a significant amount of value to the paper. It is suggested that it either be explained further in the document, simplified, or removed.

 Response:

 To avoid misunderstanding, Figure 4 has been removed and will be included in a subsequent paper.

 19. On page 12, paragraph beginning with "Owing to their good ability..." the term "global optimization" seems to give the impression that metaheuristics provide optimal solutions.

 Response:

 A clarification has been added: “However, these models still need improvement to provide truly optimal solutions.”

 20. A few citations are included in this review for the authors' consideration.

 Response:

 All suggested citations have been incorporated to enhance the paper's quality.

 Thank you for providing them.

 Thanks

 Best Regards

 Moise

# Response

**Published:** 2025-01-31

**Authors:** Moise KAMBALA MALUNDAMENE

**Affiliations:** Mines, Ecole Nationale Superieure des Mines de Rabat, Agdal, Rabat-Sale-Zemmour-Zaer, Morocco

Hello Andrea,

 Happy new year, Thanks for your time to review our paper.

 Please, See below our responses to your comments

 1. It is suggested that the authors add "surface" or "open-pit" to the title of the paper.

**Response: **We thank the reviewer for the valuable insight to help us improve the content. The title has been updated to “State-of-the-art Optimization Methods of Open-Pit Short-Term Mine Planning” to reflect the focus on open-pit mining.

 2. In the abstract, the statement "short-term planning is more challenging because it deals with uncertainties" is vague and does not reflect the actual reality that all planning time horizons deal with uncertainties.

**Response: **The sentence has been revised for clarity and context to: “Short-term planning deals with daily challenges, such as variations in block models and mining cut polygons.”

 3.There are numerous grammatical errors and typos throughout the document. For example, mining planning is used in a few places. The proper term would be mine planning.

**Response: **The document has been reviewed thoroughly to correct grammatical errors and inconsistencies. The term “mine planning” has been used consistently throughout.

 4. The introduction should make it clear that this paper is documenting the current state of open-pit or surface short-term mine planning.

**Response:** The introduction has been updated to explicitly state that the paper focuses on the current state of open-pit short-term mine planning.

 5. Review the document to ensure that descriptions are using appropriate adjectives and not colloquial terms.

**Response: **Appropriate adjectives have been used, and colloquial terms have been removed to maintain a formal tone.

 6. Inconsistent capitalization and formatting in numerous areas. See Table 2 for some examples.

**Response: **All capitalization and formatting inconsistencies have been corrected, including those in Table 2.

 7. Some figures are too small to read, e.g., Figure 1.

**Response:** The text size in Figure 1 has been increased for better readability.

 8. There are commercially available, heuristic-based scheduling solutions, e.g., RPM Global's XPAC.

**Response:** A discussion of heuristic-based scheduling solutions, including RPM Global’s XPAC, has been integrated into the conclusion.

 9. In some sections, you refer to a geologic model as a "bloc model" and in other areas as a "block model." I believe the industry standard is "block model."

 Response:

 The term “block model” has been consistently applied throughout the document.

 10. Please make sure to use commas when listing more than three items.

 Response:

 Commas have been correctly applied in lists containing more than three items.

 11. Define SMU in the text.

 Response:

 The term SMU (Small Mining Unit) has been defined in the text.

 12. It should be made clear that short-term solutions often determine destination. This is not explicitly stated in the paper.

 Response:

 This has been explicitly stated in Section 1.1, highlighting that short-term planning often determines material destinations.

 13. It seems that Figure 3 could be formatted to make the text larger.

 Response:

 The text in Figure 3 has been enlarged for clarity.

 14. The statement on page 9 of the PDF file states, "They used the CPLEX optimization method." This should be stated as they used CPLEX to "solve" the formulation.

 Response:

 The statement has been revised to indicate that CPLEX was used to “solve” the formulation.

 15.Review the document for proper tense. See paragraph beginning with "Upadhyay and Askari-Nasab (2018)" on page 9 as an example.

 Response:

 The grammar and tense in the document, including the mentioned paragraph, have been reviewed and corrected.

 16. Section 4.2, first paragraph, should make it clear that these solutions are for "Strategic" mine planning.

 Response:

 The first paragraph of Section 4.2 has been updated to clarify that the solutions discussed pertain to strategic mine planning.

 17. The authors abbreviate branch-and-bound as BB, but then use the term as B&B.

 Response:

 The abbreviation has been standardized as “B&B” throughout the paper.

 18. Figure 4 does not seem to provide a significant amount of value to the paper. It is suggested that it either be explained further in the document, simplified, or removed.

 Response:

 To avoid misunderstanding, Figure 4 has been removed and will be included in a subsequent paper.

 19. On page 12, paragraph beginning with "Owing to their good ability..." the term "global optimization" seems to give the impression that metaheuristics provide optimal solutions.

 Response:

 A clarification has been added: “However, these models still need improvement to provide truly optimal solutions.”

 20. A few citations are included in this review for the authors' consideration.

 Response:

 All suggested citations have been incorporated to enhance the paper's quality.

 Thank you for providing them.

 Thanks

 Best Regards

# Reviewer report: Reviewer response for version 1

**DOI:** 10.5256/f1000research.167816.r331455

**Published:** 2024-11-08

**Authors:** Chengkai Fan

**Affiliations:** 1 Université Laval, Québec, Canada

1. The authors list a number of papers that have provided an review of short-term or long-term planning. What is the significance of this review for the author? What is the difference from previous studies? I suggest adding the specific differences in Table 1.

 2. Please add explanations and differences between short-term, medium-term as well as long-term plans. Is there a quantitative range that distinguishes these three terms?

 3. Please spell out the abbreviations when they first appear. For example, RM and UG. 

 4. The research gap is not clear to me. Can you clarify your research gap of this review paper? Not the research gap of computational techniques. 

 5. Please explain why this review paper focuses on the two aspects of short-term planning.

 6. Authors need to summarize previous research and offer constructive comments or ideas. For example, the author overviews the application of clustering methods. The authors simply read the relevant literature and methods without making some summarizing conclusions and recommendations. This problem also applies to other parts of the review.

Is the review written in accessible language?

Yes

Are all factual statements correct and adequately supported by citations?

Yes

Are the conclusions drawn appropriate in the context of the current research literature?

Yes

Is the topic of the review discussed comprehensively in the context of the current literature?

Partly

Reviewer Expertise:

Open pit mining, machine learning, mine transport, and rock mechanics.

I confirm that I have read this submission and believe that I have an appropriate level of expertise to confirm that it is of an acceptable scientific standard, however I have significant reservations, as outlined above.

# Response

**Published:** 2025-01-15

**Authors:** Moise KAMBALA MALUNDAMENE

**Affiliations:** Mines, Ecole Nationale Superieure des Mines de Rabat, Agdal, Rabat-Sale-Zemmour-Zaer, Morocco

Hello Chengkai Fan,

 Happy new year,

 Thanks for your time to review our paper.

 Please, See below our responses to your comment

 1. The authors list a number of papers that have provided a review of short-term or long-term planning. What is the significance of this review for the author? What is the difference from previous studies? I suggest adding the specific differences in Table 1.

**Response: **We thank the reviewer for the shrewd comments and observations. Table 1 highlights the limited number of review papers focused solely on short-term planning for open-pit mines. Unlike Fathollahzadeh et al. (2021), which summarizes both underground and open-pit reviews with a focus on monthly to yearly planning, this review specifically emphasizes short-term mine planning with a daily to monthly granularity. To enhance clarity, we added a new column to Table 1 indicating the time discretization granularity for each reviewed study.

 2. Please add explanations and differences between short-term, medium-term, and long-term plans. Is there a quantitative range that distinguishes these three terms?

**Response: **We have added an explanation detailing the connections and distinctions between long-term, medium-term, and short-term planning horizons. Figure 1 has been updated to clearly illustrate these differences. Short-term planning typically spans from daily to monthly (up to three months, depending on the organization), medium-term planning covers 3 months to 3 years, and long-term planning exceeds 3 years, focusing on the life-of-mine perspective.

 3. Please spell out the abbreviations when they first appear. For example, RM and UG.

**Response:**

 All abbreviations have been defined in the text. For instance, RM stands for Resource Modeling, and UG refers to Underground.

 4. The research gap is not clear to me. Can you clarify your research gap of this review paper? Not the research gap of computational techniques.

**Response: **We have clarified the research gap in the corresponding section. This review highlights that short-term mine planning optimization for open-pit mines, with a granularity of daily to monthly, remains underexplored. Many of the papers listed in Figure 1 focus on short-term planning with time discretization extending up to one year.

 5. Please explain why this review paper focuses on the two aspects of short-term planning.

**Response: **The two aspects are critical for short-term mine planning. Before planning, mining cuts must be defined based on factors such as cut-off grade, shape, and operational constraints. Defining dig limits between ore and waste ensures alignment with the long-term strategy. Once mining cuts are established using mining polygons, short-term plans can be optimized while aligning with medium- and long-term strategies.

 6.Authors need to summarize previous research and offer constructive comments or ideas. For example, the author overviews the application of clustering methods. The authors simply read the relevant literature and methods without making some summarizing conclusions and recommendations. This problem also applies to other parts of the review.

**Response:** A summary of previous research has been included in the conclusion, where we discuss the differences and advantages of each method, particularly between dig-limit optimization and clustering.

 Thanks

 Best Regards

 Moise
